# Draft Genome Sequences of Two Clinical Isolates of *Burkholderia mallei* Obtained from Nasal Swabs of Glanderous Equines in India

**DOI:** 10.1128/genomeA.00063-17

**Published:** 2017-04-06

**Authors:** Harisankar Singha, Praveen Malik, Sheetal Saini, Sandip K. Khurana, Mandy C. Elschner, Katja Mertens, Stefanie A. Barth, Bhupendra N. Tripathi, Raj K. Singh

**Affiliations:** a ICAR-National Research Centre on Equines, Hisar, India; b Friedrich-Loeffler-Institut, Federal Research Institute for Animal Health, Institute of Bacterial Infections and Zoonoses, Jena, Germany; c Friedrich-Loeffler-Institut, Federal Research Institute for Animal Health, Institute of Molecular Pathogenesis, Jena, Germany

## Abstract

*Burkholderia mallei* is a Gram-negative coccobacillus which causes glanders—a fatal disease of equines that may occasionally be transmitted to humans. Several cases of outbreaks have been reported from India since 2006. This paper presents draft genome sequences of two *B. mallei* strains isolated from equines affected by glanders in India.

## GENOME ANNOUNCEMENT

Glanders is a fatal and contagious disease of equines caused by the bacterium *Burkholderia mallei*. The disease in equines is characterized by chronic suppurative lesions of skin and mucous membrane, pneumonia, and septicemia (http://www.oie.int/index.php?id=169&L=0&htmfile=chapitre_glanders.htm). Asymptomatic carrier horses are the only natural reservoirs of *B. mallei* and the source of infection. It is a notifiable disease to the World Organization of Animal Health (OIE) and the organism is classified as category B select agents by the U.S. Centers for Disease Control and Prevention (1). Unprecedented emergence of glanders in several countries throughout the Middle East, Southern Asia, parts of Africa, and South America were reported in the past decade (2). Following the re-emergence of glanders in northern states of India during 2006, the disease has spread to several states of India over the past decade affecting hundreds of equines (3). Here, we present the draft genome sequences of two *B. mallei* strains 3076HP_India and 3712UP_India, which were isolated from clinical samples (nasal swab) of a mule and a mare in 2013 and 2015, respectively.

Genomic DNA was extracted from stationary phase *B. mallei* culture using the Gene Jet DNA purification kit (Thermo Scientific, USA). The bacterial genome was sequenced on an Illumina HiSeq 2500 using 2 × 125 bp chemistry at Eurofins Genomics (India). The raw reads generated were filtered using Trimmomatic v0.35 with quality value QV > 30. The filtered high-quality reads were assembled using Velvet v1.2.10 (4). Genes were predicted from the assembled sequences using Prodigal v2.6.3 with default parameters (5). Functional annotation of the genes was performed using NCBI BLASTx-2.3.0+ stand alone tool using the nonredundant (NR) protein database (https://blast.ncbi.nlm.nih.gov/Blast.cgi?PROGRAM=blastx&PAGE_TYPE=BlastSearch&BLAST_SPEC=&LINK_LOC=blasttab&LAST_PAGE=blastn). The assembled genomes were aligned to the *B. mallei* ATCC 23344 reference genome using MUMmer v3.0 with default parameters and a circos plot was generated for graphical representation using Circos v0.69.1 (6, 7).

The high-quality data thus obtained were 2.2 GB with 8,810,805 reads. Genome assembly generated 250 and 257 contigs (215 scaffolds) with an *N*_50_ of 36,813 bp for the *B. mallei* strains 3076_HP and 3712_UP, respectively. The assembled genome was 5.6 Mb in size with 67.07% G+C content. A total number of 4,713 genes were predicted with an average gene size of 987 bp. In annotation, 4,660 genes found hits while 53 genes were not annotated against the NR database. More genomic data of clinical *B. mallei* isolates from different geographical locations may be useful in providing insight into the molecular evolution and epidemiology.

### Accession number(s).

This whole-genome shotgun sequencing project has been deposited at DDBJ/EMBL/GenBank under the accession numbers MDTH00000000 and MDJV00000000. The versions described in this paper are the first versions.

## References

[1] Rotz LD, Khan AS, Lillibridge SR, Ostroff SM, Hughes JM 2002 Public health assessment of potential biological terrorism agents. Emerg Infect Dis 8:225–230. https://wwwnc.cdc.gov/eid/article/8/2/01-0164_article. 11897082 10.3201/eid0802.010164 PMC2732458

[2] World Organisation for Animal Health. 2016 The world animal health information system. http://www.oie.int/animal-health-in-the-world/the-world-animal-health-information-system/the-oie-data-system/.

[3] Malik P, Singha H, Goyal SK, Khurana SK, Tripathi BN, Dutt A, Singh D, Sharma N, Jain S 2015 Incidence of *Burkholderia mallei* infection among indigenous equines in India. Vet Rec Open 2:e000129. doi:10.1136/vetreco-2015-000129. 26457190 PMC4594314

[4] Zerbino DR, Birney E 2008 Velvet: algorithms for de novo short read assembly using de Bruijn graphs. Genome Res 18:821–829. doi:10.1101/gr.074492.107. 18349386 PMC2336801

[5] Hyatt D, Chen GL, Locascio PF, Land ML, Larimer FW, Hauser LJ 2010 Prodigal: prokaryotic gene recognition and translation initiation site identification. BMC Bioinformatics 11:119. doi:10.1186/1471-2105-11-119. 20211023 PMC2848648

[6] Kurtz S, Phillippy A, Delcher AL, Smoot M, Shumway M, Antonescu C, Salzberg SL 2004 Versatile and open software for comparing large genomes. Genome Biol 5:R12. doi:10.1186/gb-2004-5-2-r12. 14759262 PMC395750

[7] Krzywinski M, Schein J, Birol I, Connors J, Gascoyne R, Horsman D, Jones SJ, Marra MA 2009 An information aesthetic for comparative genomics. Genome Res 19:1639–1645. doi:10.1101/gr.092759.109. PMC2752132 19541911

